# Integrated Analysis and Identification of Novel Biomarkers in Parkinson’s Disease

**DOI:** 10.3389/fnagi.2018.00178

**Published:** 2018-06-18

**Authors:** Jieshan Chi, Qizhi Xie, Jingjing Jia, Xiaoma Liu, Jingjing Sun, Yuanfei Deng, Li Yi

**Affiliations:** ^1^Department of Neurology, Peking University Shenzhen Hospital, Shenzhen, China; ^2^Department of Clinical Medicine, Shantou University Medical College, Shantou, China; ^3^National Clinical Research Center for Geriatric Diseases Shenzhen Center, Peking University Shenzhen Hospital, Shenzhen, China

**Keywords:** Parkinson’s disease, bioinformatics, biomarkers, genes, miRNA, NAFD pathway

## Abstract

Parkinson’s disease (PD) is a quite common neurodegenerative disorder with a prevalence of approximately 1:800–1,000 in subjects over 60 years old. The aim of our study was to determine the candidate target genes in PD through meta-analysis of multiple gene expression arrays datasets and to further combine mRNA and miRNA expression analyses to identify more convincing biological targets and their regulatory factors. Six included datasets were obtained from the Gene Expression Omnibus database by systematical search, including five mRNA datasets (150 substantia nigra samples in total) and one miRNA dataset containing 32 peripheral blood samples. A chip meta-analysis of five microarray data was conducted by using the metaDE package and 94 differentially expressed (DE) mRNAs were comprehensively obtained. And 19 deregulated DE miRNAs were obtained through the analysis of one miRNAs dataset by Qlucore Omics Explorer software. An interaction network formed by DE mRNAs, DE miRNAs, and important pathways was discovered after we analyzed the functional enrichment, protein–protein interactions, and miRNA targetome prediction analysis. In conclusion, this study suggested that five significantly downregulated mRNAs (MAPK8, CDC42, NDUFS1, COX4I1, and SDHC) and three significantly downregulated miRNAs (miR-126-5p, miR-19-3p, and miR-29a-3p) were potentially useful diagnostic markers in clinic, and lipid metabolism (especially non-alcoholic fatty liver disease pathway) and mitochondrial dysregulation may be the keys to biochemically detectable molecular defects. However, the role of these new biomarkers and molecular mechanisms in PD requires further experiments *in vivo* and *in vitro* and further clinical evidence.

## Introduction

Parkinson’s disease (PD) is a quite common neurodegenerative disorder with a prevalence of approximately 1:800–1,000 in subjects over 60 years of age (Bekris et al., 2010; Lin and Farrer, 2014). The pathological hallmarks of PD are the degeneration of dopaminergic neurons in the substantia nigra (SN) pars compacta and Lewy bodies formed by the accumulation of α-synuclein (SNCA) in the remaining neurons (Spillantini et al., 1997; Braak et al., 2003). PD is a progressive disease characterized by motor signs such as resting tremor, bradykinesia, muscle rigidity, and posture abnormalities, but though in some patients, non-motor symptoms may appear, including depression, anxiety, sleep disturbance, fatigue, cognitive disorders, and gastrointestinal and sexual dysfunction (Schneider and Obeso, 2015).

Parkinson’s disease was originally thought to be a sporadic disease caused by environmental factors such as exposure to 1-methyl-4-phenyl-1,2,3,6-tetrahyropyridine (MPTP) or paraquat (Langston et al., 1983; Ascherio and Schwarzschild, 2016). However, epidemiological investigation in recent decades found that approximately 10% of patients had a family history of PD, and approximately 90% of patients had sporadic PD (Klein and Westenberger, 2012; Ascherio and Schwarzschild, 2016). It is generally accepted that PD is a multifactorial disease caused by the interaction between environmental and genetic factors, and approximately 5–10% of PD cases have a mutation in one of the several disease-associated genes according to the report (Farrer, 2006; Kalia and Lang, 2015). Several genes have been shown to be closely related to PD, such as α-synuclein (SNCA), leucine-rich repeat kinase 2 (LRRK2), Parkin RBRE3 ubiquitin protein ligase (PRKN), Parkinsonism associated deglycase (PARK7), PTEN-induced putative kinase 1 (PINK1), and ATPase 13A2 (ATP13A2) (Bekris et al., 2010; Nuytemans et al., 2010; Klein and Westenberger, 2012; Heman-Ackah et al., 2013). However because these mutations do not cause disease directly, it is more likely that they make people more susceptible to the PD when in cooperation with other risk factors (Lesage and Brice, 2009). In sporadic PD patients, SNCA and mitochondrial dysfunction are the predominant components of Lewy bodies, and complex I is found to be defective in the cytoplasm in the SN (Henchcliffe and Beal, 2008). However, what we know about the potential mechanism of PD is just the tip of the iceberg. At the genomic level, it is rather difficult to analyze the disease, especially sporadic PD, from a single gene mutation, and it is more the imbalance in gene expression and phenotypic changes were caused by a variety of regulatory mechanisms which act as mediators between genotype and phenotype. microRNA (miRNA) is considered as one of these mediators. Some deregulatory miRNAs were detected in the humans that lead to mitochondrial dysfunction, altered mitochondrial dynamics, oxidative stress, excitotoxicity, and the accumulation of SNCA, consequently resulting in neurodegeneration (Martin, 2010). Since miRNAs and its target genes are not in a one-to-one but one-to-many relationship (Qiu et al., 2015), the mutual regulation network between miRNAs and target genes can provide more insight into the disease and possible new treatments. Although the exploration of PD has been explored for nearly a century, the pathogenesis of PD is not yet clear.

Massively parallel microarray analysis allows for the global assessment of more credible relationships between gene expression and clinical manifestations in unbiased, and reveals the etiology of such complex diseases by identifying abnormalities in genes or pathways (Schadt et al., 2005; Scherzer, 2009). Large-scale genetic data can be categorized and detected based on phenotypic characteristics and produce hypotheses about the mechanisms of disease, which may have an underestimated role in the decoding of complex diseases (Scherzer et al., 2008). The utility of genome-wide expression data is often subjected to typical inconsistent analysis, non-replication, and small sample effects in practice.

To effectively reducing the bias of small sample studies and nominating deregulated genes, this integrated analysis consolidated data information on multiple datasets from different platforms. The aim of our work was to determine the candidate target genes in PD through meta-analysis and bioinformatic analysis of multiple datasets in gene expression and to further combine mRNA and miRNA expression analyses to seek out more convincing biological targets and their regulatory factors. In this study, we found several new potential mRNAs and miRNAs as well as one pathway in combination with the mRNA and miRNA microarray analysis and have mapped the intrinsic roles of genes and pathways in PD.

## Materials and Methods

### Materials and Data Pre-processing

Candidate microarrays associated with PD were acquired by retrieving the human gene expression studies deposited in the Gene Expression Omnibus (GEO) database^1^. Datasets related to PD were researched with the term “PD” of *Homo sapiens* (target). By September 14, 2017, a total of 1418 datasets were retrieved, including different types of samples and various types of expression data. The inclusion criteria were as follows: (1) original experimental studies; (2) human SN sample; (3) mRNA expression profile; (4) can obtain the unprocessed raw data (CEL files). The exclusion criteria were as follows: (1) repeated reports from the same institute or hospital; (2) non-human SN sample; (3) a non-expression gene chip, and the unprocessed raw data (CEL files) of these datasets were acquired from GEO database. All the included studies obtain relative ethics approval. All datasets were pre-processed individually (including background adjustment, normalization, summarization) on the base-2 logarithm by robust multi-array average (RMA) and annotated by converting different probe IDs to gene IDs by using R language. We use the Bioconductor software to compute RMA expression measures. Loaded the appropriate software with library(affy) to read all the CEL files in the current working directory. After loading the data, we compute the RMA expression measure. For miRNA microarray, the unqualified chips would be retrieved, and the samples for RNA detection were human peripheral blood. The miRNA dataset was imported into the Qlucore Omics Explorer (QOE) software for data pre-processing (mean = 0, SD = 1).

### Integrated Analysis of Gene Expression Datasets

Included gene expression datasets were loaded into R language for objective quality control by MetaQC package, which intended to identify whether the included chip was qualified for genomic meta-analysis. The MetaQC package provides four quantitative quality control indexes, including internal quality control (IQC), external quality control (EQC), accuracy quality control of differentially expressed (DE) gene detection (AQCg), or pathway identification (AQCp) and consistency quality control in genes (CQCg) or pathways (CQCp). A principal component analysis (PCA) was performed to further visualize the quality control results. Eligible microarrays were subjected to threshold screening individually to obtain DE genes in PD under specific conditions using the Linear Models for Microarray (LIMMA) package. For integrated analysis, we further carried out the genomic meta-analysis. Considering the feasibility of the methods, a modified two-sample *t*-test by adding a fudging parameter was used to extrapolate the *P*-values and Fisher’s method was implemented for statistical analysis of significance (Tseng et al., 2012). A corrected *P*-value (*P* < 0.05) was considered statistically significant for the DE mRNAs. For visualization, DE mRNAs based on specific fault discovery rate (FDR) values (FDR < 0.0001) were plotted by the MetaDE package (heatmap.sig.genes).

### Functional Analysis of PD-Related DE Genes

To access the prospective functions of PD-related DE genes found in the meta-analysis, online tools such as the Database for Annotation, Visualization and Integrated Discovery (DAVID) (Huang da et al., 2009a,b) were used. The functional categories Kyoto Encyclopedia of Genes and Genomes (KEGG) pathways and Gene Ontology (GO) terms were analyzed. For narrated KEGG pathways and GO terms that enriched the target genes, *P*-values less than 0.05 were defined as the cut-off criterion. The critical assessment and integration of protein–protein interactions (PPIs) of PD-related DE genes, including direct as well as indirect associations, were analyzed by the STRING database (Szklarczyk et al., 2015). The PPI networks were visualized by Cytoscape software (Shannon et al., 2003).

### Analysis of miRNA Expression Dataset and Target Prediction

Qlucore Omics Explorer software was primarily capable of screening for DE miRNAs (Jimbo et al., 2011). Primarily, QOE software performed the normalized processing of mean = 0 and var = 1 for the original data, and further calculated the corresponding standard error σ for each variable. We filtered out the variables with small differences among samples according to the statistics by σ/σ_max_. After data preprocessing, the comparison between two groups was performed and specific filter variable parameters were selected for statistical analysis of significance (*P* < 0.001, *q* < 0.01, fold change > 2). The predicted target genes of DE miRNAs were identified by three different target prediction algorithms: miRDB^2^ (Wong and Wang, 2015) TargetScan 7.1^3^ (Agarwal et al., 2015) and microT_CDS of Diana Tools^4^ (Reczko et al., 2012; Paraskevopoulou et al., 2013). Unique genes with target sites on 3′-UTR were incorporated. To reduce the false positive rate and improve persuasion, we obtained the overlap target genes of the three algorithms mentioned above.

## Results

### Integrated Analysis of PD Gene Expression Datasets

**FIGURE 1:**
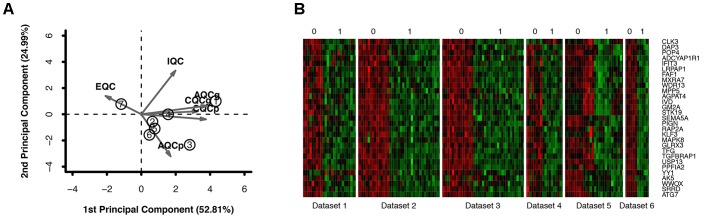
**(A)** Quality control result. **(B)** DE genes through the method of meta-DE. (For the sake of space, only a part of the figure is shown here. The full size figure is displayed in Online Resource **Supplementary Figure S1**.

Six primary datasets with available mRNA expression data for SN samples in PD patients and controls were identified by searching the GEO database (GSE20186, GSE8397, GSE20141, GSE20333, GSE7621, and GSE20295). One of these eligible datasets (GSE20295) was excluded after the quality control using the MetaQC package and visualization by PCA (**Figure 1A**, additional data were given in Online Resource **Supplementary Table S1**). The remaining five original datasets (accession numbers GSE20186, GSE8397, GSE20141, GSE20333, and GSE7621), six microarrays in total (dataset GSE8397 contained two mRNA chips: 39 individual tissue samples were tested using one A and one B mRNA chip per sample), were incorporated in our meta-analysis, for a total of 150 independent SN samples (80 PD patients and 70 controls, **Table 1**). Each dataset was analyzed independently to obtain its own DE mRNAs before performing pooled analysis. Approximately 193 DE mRNAs (hereinafter referred to as overlap DE mRNAs) were confirmed to co-exist in all eligible datasets. After merging the datasets, we identified 94 mRNAs (hereinafter referred to as meta-DE mRNAs) showing consistent DE patterns using a penalized *t*-test by adding a fudging parameter, and the maximum *P*-value and Fisher’s method by summarizing -log (*P*-value) across studies were chosen to eliminate the significant influence of the large number of samples (Lu et al., 2010). The heatmap of the meta-DE mRNAs was plotted and visualized by setting a particular FDR (<0.00001, **Figure 1B** and additional data were given in Online Resource **Supplementary Figure S1**), which demonstrated that meta-DE mRNAs were all downregulated (meta-DE mRNAs expression in each dataset are shown in Online Resource **Supplementary Table S2**, and the details of each meta-DE mRNA are given in Online Resource **Supplementary Table S3**).

**Table 1:** Baseline characteristics of datasets.

Study	Country	GEO accession	Platform ID	Sample type	Experiment type	Cases/controls		
						Number	Age at death (range)	Male (%)
Zheng	USA	GSE20186	GPL96	SN^a^	mRNA	14/14	70–84/52–90	∖
Moran	UK	GSE8397-A	GPL96	SN	mRNA	24/15	68–89/46–81	66.6/58
		GSE8397-B	GPL97					
Middleton	USA	GSE20141	GPL570	SN	mRNA	10/8	63–93/47–94	50/38
Jasmine	Israel	GSE20333	GPL201	SN	mRNA	6/6	70–87/68–88	67/67
Mullen	USA	GSE7621	GPL570	SN	mRNA	16/9	63–87/46–90	81/44
Middleton	USA	GSE20295	GPL96	SN	mRNA	10/18	70–82/41–94	60/61
Oliveira	Portugal	GSE16658	GPL7722	PB^b^	microRNA	19/13	58–71/51–73	53/38

*^a^Substantia nigras. ^b^Peripheral blood.*

### Enrichment Analysis of the DE Gene

**FIGURE 2:**
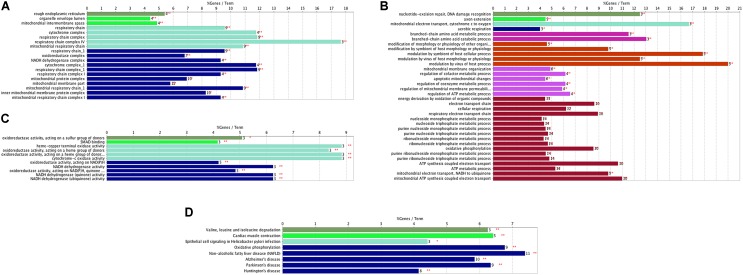
Functional enrichment analysis of meta-DE genes. **(A)** Cellular components of GO enrichment analysis. **(B)** Biological processes of GO enrichment analysis. **(C)** Molecular functions of GO enrichment analysis. **(D)** KEGG pathway enrichment analysis.

Through the enrichment analysis of KEGG pathways, meta-DE genes were mainly involved in the Huntington’s disease pathway, PD pathway, Alzheimer’s disease pathway, non-alcoholic fatty liver disease (NAFLD) pathway, and the oxidative phosphorylation pathway (**Figure 2D**). The results of enrichment analysis using three categories of GO were as follows: (1) biological processes: mitochondrial ATP synthesis-coupled electron transport, mitochondrial membrane organization, modulation by virus of host process, branched-chain amino acid catabolic process, aerobic respiration, etc. (**Figure 2A**); (2) molecular functions: oxidoreductase activity acting on NAD(P)H, oxidoreductase activity of a heme group of donors, SMAD binding, and oxidoreductase activity of a sulfur group of donors (**Figure 2B**); and (3) cellular component: mitochondrial respiratory chain, respiratory chain complex, organelle envelope lumen, and rough endoplasmic reticulum (**Figure 2C**). The enrichment analysis by GO revealed that DE genes were mainly involved in biological processes including the ATP metabolic process and electron transfer, and the main organelles in these processes were mitochondria, which were consistent with the results of GO enrichment with cellular components.

**FIGURE 3:**
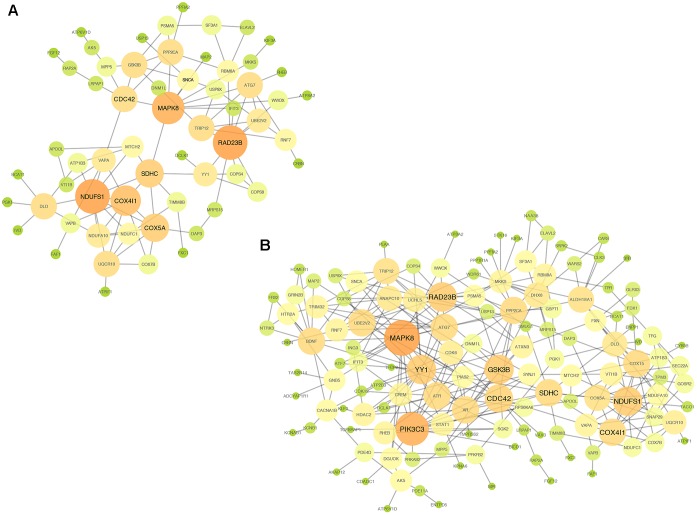
PPI networks. **(A)** PPI network of overlap DE genes. **(B)** PPI network of meta-DE genes. The size and color of map nodes are determined by the degree value, which renders a gradual process by setting the small size with a low degree in green, large size with a high degree in orange.

In addition, we established PPI networks using the STRING database to further investigate the functional partnership and interaction of overlapping DE genes (**Figure 3A**) and meta-DE genes (**Figure 3B**), respectively, and the results were visualized by Cytoscape software. Interestingly, *MAPK8, CDC42, NDUFS1, COX4I1, RAD23B*, and *SDHC*, respectively, were in the core position of two PPI networks. The significant level of these six core genes was showed in **Table 2**. Once these hub nodes were expurgated, both PPI networks became inattentive. Of these core genes, *NDUFS1, COX4I1*, and *SDHC*, were significantly linked to mitochondrial function which means these three DE genes were potentially related to PD. Furthermore, in addition to the known pathways, we found a pathway (NAFLD pathway) that was not previously reported to be closely associated with PD. Of note, two of these core genes (*MAPK8, CDC42*) were significantly involved in the NAFLD pathway.

**Table 2:** The significant level of six core genes.

metaDE	GSE2033		GSE8397-A		GSE8397-B		GSE20141		GSE20186		GSE7621	
Genes	logFC	adj.P.Val	logFC	adj.P.Val	logFC	adj.P.Val	logFC	adj.P.Val	logFC	adj.P.Val	logFC	adj.P.Val
CDC42	-0.663665756	0.001848206	-1.354477736	1.26765E-19	-0.52505805	0.001771286	-1.520805162	0.004946437	-0.854236199	0.0000725	-0.880281614	0.003490547
MAPK8	-1.033036228	0.000159255	-0.614815263	1.46E-13	-1.164926551	1.36E-22	-1.518136177	0.000202715	-1.518136177	0.000202715	-1.552806077	6.68E-08
NDUFS1	-1.465304476	0.000128654	-1.264918058	4.68E-14	-0.475942645	4.68E-14	-1.582731583	0.007653762	-0.806936955	0.003515467	-0.406495108	0.046279673
COX4I1	-1.030593768	2.48176E-05	-0.495018251	2.62E-09	-0.490606125	2.62E-09	-1.002486741	0.012615617	-0.574023971	0.0000484	-0.498111013	0.00000193
RAD23B	-1.144208007	0.001236886	-0.526176438	4.33E-08	-1.108709011	4.33E-08	-1.484149567	0.0000855	-0.469560145	0.017881861	-0.611753774	0.000172396
SDHC	0.000563161	-0.902524575	-0.659662639	1.91E-10	-0.713186333	1.44E-16	-1.325925284	0.003084895	-0.511124126	2.82E-05	-0.510356689	1.18E-05

### Analysis of the PD miRNA Expression Dataset

**FIGURE 4:**
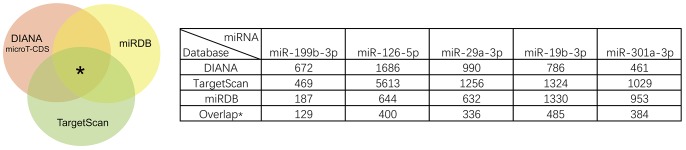
Summary results of the consensus of targets genes of top five miRNAs.

We identified the GSE16658 dataset with available miRNA expression data containing 32 peripheral blood samples of PD patients (19 samples) and controls (13 samples) by searching the GEO database. Nineteen DE miRNAs were identified using QOE software; among them, the top five most significant DE miRNAs were miR-199-3p (miR-199a-3p/miR-199b-3p), miR-126-5p, miR-29a-3p, miR-19b-3p, and miR-301a-3p (**Table 3**). The miRNA target genes were obtained from experimentally supported databases with experimentally verified and different prediction algorithms. The consensus of the target genes of the top five miRNAs was summarized (**Figure 4**, additional data are provided in Online Resource **Supplementary Table S4**). Here, we identified 11 genes coexisted in the union set of all target genes and meta-DE mRNAs (*NDUFS1, MAPK8, CDC42, SNCA, VAPA, USP13, TIMM8B, KIF3A, KPNA6, MTCH2*, and *SUB1*).

**Table 3:** The DE miRNAs.

miRNA ID	Mature miRNA	*P*-value	Fold change	Sequence
hsa-miR-199b-3p	miR-199b-3p	3.14E-05	2.18189	UAGCACCAUUUGAAAUCGGUUA
hsa-miR-126-5p	miR-126-5p	4.59E-05	3.01653	CAGUGGUUUUACCCUAUGGUAG
hsa-miR-29a	miR-29a-3p	0.000180049	2.01085	UGAGGUAGUAGAUUGUAUAGUU
hsa-miR-19b	miR-19b-3p	0.0001859	2.4704	UAUUGCACAUUACUAAGUUGCA
hsa-miR-301a	miR-301a-3p	0.000211687	2.47262	UGAGGUAGUAGUUUGUGCUGUU
hsa-miR-19a	miR-19a-3p	0.000280255	2.60849	UGUGCAAAUCUAUGCAAAACUGA
hsa-miR-142-5p	miR-142-5p	0.000282505	2.14868	UGUAAACAUCCUUGACUGGAAG
hsa-miR-101	miR-101-3p	0.000402333	2.63582	UAAGGUGCAUCUAGUGCAGUUAG
hsa-miR-30e	miR-30e-5p	0.000508645	2.82353	UAAGGUGCAUCUAGUGCAGAUAG
hsa-miR-140-5p	miR-140-5p	0.00069842	2.20528	UGUAGUGUUUCCUACUUUAUGGA
hsa-let-7g	let-7g-5p	0.000811595	2.22691	CAUUAUUACUUUUGGUACGCG
hsa-miR-142-3p	miR-142-3p	0.00086	2.55601	UAGCACCAUUUGAAAUCAGUGUU
hsa-miR-105	miR-105-5p	0.000997507	2.24747	ACAGUAGUCUGCACAUUGGUUA
hsa-let-7f	let-7f-5p	0.00123097	2.06713	UGAGGUAGUAGUUUGUACAGUU
hsa-miR-32	miR-32-5p	0.00138919	2.43632	CAUAAAGUAGAAAGCACUACU
hsa-miR-18b	miR-18b-5p	0.0014553	2.15171	UAGCUUAUCAGACUGAUGUUGA
hsa-miR-18a	miR-18a-5p	0.00163672	2.1271	UCAAAUGCUCAGACUCCUGUGGU
hsa-let-7i	let-7i-5p	0.00175562	2.10813	UACAGUACUGUGAUAACUGAA
hsa-miR-21	miR-21-5p	0.00226262	2.47414	UGUGCAAAUCCAUGCAAAACUGA

### Comprehensive Analysis of DE Genes and miRNAs

**FIGURE 5:**
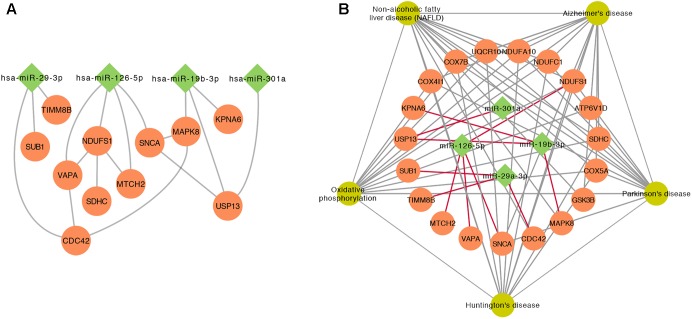
The interaction between miRNAs and genes. **(A)** The relationship of gene–gene and gene–miRNA. **(B)** The outline of the interaction between the significant KEGG pathways of meta-DE genes and miRNAs.

To further investigate the relationship between top 5 DE miRNAs and 11 DE mRNAs mentioned above, we plotted their regulatory networks by Cytoscape. The results showed that the interaction between miRNAs and genes was formed with miR-126-5p, miR-29a-3p, and miR-19b-3p as the center (**Figure 5A**). Each miRNA interacted with several meta-DE genes, including miR-126-5p with *MTCH2, VAVP, SNCA*, and *NDUFS1*; miR-29a-3p with *CDC42, TIMM8B*, and *SUB1*; and miR-19b-3p with *MAPK8, USP13*, and *KPNA6*. Here, we outline the relationship analyzed above, including genes and pathways, particularly the main underlying pathway related to PD clustering by meta-DE genes (**Figure 5B**). Altogether, we extracted the most potential biomarkers, five mRNAs and three miRNAs, as well as one new pathway from the whole analysis as a matter of priority (**Table 4**).

**Table 4:** Priority genes, related miRNAs, and functions.

Gene	Gene name	Genomic location	Related miRNA	Predicted PD-related function
*NDUFS1*	NADH: Ubiquinone oxidoreductase core subunit S1	2q33.3	miR-126-5p	Mitochondrial function
*COX4I1*	Cytochrome C oxidase subunit 4I1	16q24.1	∖	Mitochondrial function
*SDHC*	Succinate dehydrogenase complex subunit C	1q23.3	∖	Mitochondrial function
*CDC42*	Cell division cycle 42	1p36.12	miR-29a-3p	NAFLD pathway
*MAPK8*	Mitogen-activated protein kinase 8	10q11.22	miR-19b-3p	NAFLD pathway

## Discussion

We utilized a careful method to standardize and unify the cross-platform datasets of gene profiling in PD for further integrated analyses. The integrated analysis method described here aimed to reduce the bias of small sample studies and nominating deregulated genes effectively. We have included six gene expression data point from five different platforms in PD and then performed meta-analysis using the MetaDE package in R language for merging and gene filtering of gene expressions (Wang et al., 2012). We identified 94 down regulated DE mRNAs in SN samples of PD and 19 deregulated miRNAs in PB samples of PD. Of these DE mRNAs and miRNAs, we deemed five genes (*NDUFS1, COX4I1, SDHC, CDC42*, and *MAPK8*) and three miRNAs (miR-126-5p, miR-29a-3p, and miR-19b-3p) worth exploring due to their relationship with the NAFLD pathway or the biological process of mitochondrial function.

Previous studies have suggested that several miRNAs, such as miR-133b (Kim et al., 2007), miR-16 (Zhang and Cheng, 2014), miR-153 (Doxakis, 2010), miR-205 (Cho et al., 2013), miR-7 (Junn et al., 2009; Doxakis, 2010), miR-64, miR-65, and the let-7 family (Asikainen et al., 2010), were related to PD. In our research, 19 significantly DE miRNAs were identified including let-7f and let-7g (Asikainen et al., 2010), which were confirmed previously and several new miRNAs. To further verify the results of our integrated analysis in a more comprehensive way, we compared the target genes predicted by the top five DE miRNAs with the analyzed DE mRNAs, and we initially conjectured that miR-126-5p, miR-19-3p, and miR-29a-3p were more likely to participate in the pathogenesis of PD.

The enrichment analysis by GO revealed that DE genes were mainly involved in biological processes including ATP metabolism process and electron transfer, and the main organelles in these processes were mitochondria, which was consistent with the results of GO enrichment with cellular components. Mitochondrial dysfunction is closely linked to the occurrence and development of PD (Stoessl, 1999; Koyano et al., 2013). Damage to the electron transport chain, the inhibition of complex I activity, mishandling of calcium, enhanced sensitivity to mitochondrial toxins, oxidative stress, and dysfunctional mitochondrial dynamics could contribute to neuronal dysfunction and participate in the pathogenesis of PD (Schapira et al., 1990; McCoy and Cookson, 2012). As an important energy-producing organelle in cells, mitochondria oxidize carbon and further produced ATP through oxidative phosphorylation (Wallace et al., 2010). Accordingly, damage to mitochondria in any process may disrupt the energy balance and promote the occurrence of the disease. Combining the results of two PPI networks, the core genes linked to mitochondrial function included three mitochondrial-localized genes (*NDUFS1, COX4I1*, and *SDHC*).

Both *NDUFS1* and *COX4I1* are located at the mitochondrial inner membrane and play an integral role in the electron transport chain. It was noted that pyrroloquinoline quinone can protect SH-SY5Y cells from the cytotoxicity induced by a mitochondrial complex I inhibitor (Zhang et al., 2014). However, silencing *NDUFS1* in midbrain neurons or SH-SY5Y cells reduced the neuroprotective effect of pyrroloquinoline quinone (Zhang et al., 2016), which means that the down regulation of *NDUFS1* may be detrimental to mitochondrial function. As a core subunit of ubiquinone oxidoreductase, the deregulation of *NDUFS1* might result from the pathogenesis of PD by undermining mitochondrial function. In addition, production coded by *COX4I1* is the terminal enzyme of the mitochondrial respiratory chain. A large-scale study of PD gene expression profiling demonstrated that *COX4I1* was downregulated in whole-blood in patients with PD (Shamir et al., 2017). The consensus of deregulation both in blood and SN indicated that *COX4I1* may be a potential biomarker in PD. There is currently no significant evidence that *SDHC* is involved in the pathogenesis of PD. However, its coding product as a member of nuclear-encoded subunits that comprises succinate dehydrogenase (also known as mitochondrial complex II) is a key enzyme complex of the tricarboxylic acid cycle and aerobic respiratory chains of mitochondria, which shows that the relationship between *SDHC* and PD deserves further study.

Kyoto Encyclopedia of Genes and Genomes pathway enrichment analysis for meta-DE genes shows that the genes relate to the neurodegenerative disease signaling pathway, including PD, Alzheimer’s disease and Huntington’s disease, and the NAFLD pathway. Neurodegenerative diseases share fundamental processes, such as mitochondrial anomalies and oxidative stress, in spite of their distinct pathological and clinical features (Di Carlo et al., 2012). Simultaneously, cholinergic deficit is a common pathogenesis among these three diseases and leads to analogous clinical manifestations such as dyskinesia (Pepeu and Grazia Giovannini, 2017). These prevailing conclusions support the effectiveness and validity of our integrated analysis.

Here, we proposed that the NAFLD pathway may probably be a new PD-related pathway that has been reportedly linked to neurodegeneration, especially AD. The study of APP-Tg mice indicates that NAFLD offers the opportunity to accelerate the symptoms of AD (Kim et al., 2016). A long-term high-fat diet induces systemic inflammation that stimulates the central nervous system to cause neurodegeneration (Kim et al., 2016). Studies that explore the association of NAFLD with PD are currently lacking, however, evidence from clinical and experimental investigations is mounting that alterations in lipid metabolism participate in the pathogenesis through direct crosstalk between lipids and SNCA (Ruiperez et al., 2010). Noticeably, two meta-DE genes (*MAPK8* and *CDC42*) (Sharma et al., 2012; Wang et al., 2016), including their related miRNAs (miR-19b-3p and miR-29a-3p, respectively), participate in the NAFLD pathway. An *in vivo* and *in vitro* study confirms that miR-29 can significantly inhibit HMGCR expression by targeting 3′-UTR of HMGCR mRNA and participate in the accumulation of free cholesterol in the livers of mice with non-alcoholic steatohepatitis (Liu et al., 2017). Therefore, miR-29 may be an important regulator of hepatic cholesterol homeostasis and a potential therapeutic target for the treatment of NAFLD and other liver diseases associated with free cholesterol accumulation. According to our integrated analysis, it is reasonable to assume that miR-29a-3p is involved in the development and progression of PD through the expression of *MAPK8* in the NAFLD pathway. Paradoxically, the inhibition of the *MAPK8* (also named *JNK1*) was reported to be a neuroprotective factor (Busquets et al., 2017) which seems contradictory to our analytic results. The reasons for this phenomenon are obscure and need further verification and exploration. Some reports have illustrated the role of *CDC42* in the pathophysiology of inherited PD (Musilli et al., 2016), and similarly, *CDC42* is deregulated in NAFLD (Wang et al., 2016) which may significantly indicate the pivotal role of *CDC42* in PD especially though the potential NAFLD pathway. Its related miRNA (miR-19b-3p) may participate in neurodegeneration by modulating neural cell apoptosis (Wang et al., 2016). We hereby propose that it is meaningful to conduct in-depth follow-up studies on the relationship between the NAFLD pathway and PD to elucidate the pathogenesis of PD and find new therapeutic targets or preventive measures.

This study has several limitations. One is the limited number of included datasets, especially the miRNA datasets. The other is the limited sample size. In the preliminary protocol, we intended to have brain, blood, and cerebrospinal fluid samples included in the mRNA and miRNA microarray. However, some of these sample types were not included in the current study due to the insufficiency of datasets and the inaccessibility of the raw data. Therefore, we will monitor this progress in PD. Further investigations are required to confirm whether our new biomarkers are potential prognostic predictors or therapeutic targets in PD.

In summary, our integrated analysis of PD genomics provides us with a wealth of resources to explore the role of target genes and miRNAs in PD. Five significantly downregulated genes (MAPK8, CDC42, NDUFS1, COX4I1, and SDHC) and three significantly downregulated miRNAs (miR-126-5p, miR-19-3p, and miR-29a-3p) were potentially useful clinical diagnostic markers. Lipid metabolism (especially NAFLD pathway) and mitochondrial dysregulation may underlie biochemically detectable deeper molecular defects. Future work should be focused on these two aspects to reveal the pathogenesis of PD and to develop new therapeutic targets for the clinical treatment of PD.

## Author Contributions

JC and LY designed the protocol. JC, QX, and JJ collected the data. JS and XL standardized the raw data. JC and YD analyzed the experimental results. JC and LY wrote the manuscript.

## Conflict of Interest Statement

The authors declare that the research was conducted in the absence of any commercial or financial relationships that could be construed as a potential conflict of interest.

## Footnotes

1 https://www.ncbi.nlm.nih.gov/geo/

2 http://www.mirdb.org

3 http://www.targetscan.org/vert_71/

4 http://diana.imis.athena-innovation.gr/DianaTools
