# Supplementary material for: Integrated Analysis and Identification of Novel Biomarkers in Parkinson’s Disease
Source: Front Aging Neurosci. 2018 Jun 18;10:178. doi: 10.3389/fnagi.2018.00178 (PMC6016006; doi:10.3389/fnagi.2018.00178)
Supplement: Supplementary file 1 [file Table_1.docx]

**Table S1. Quality Control Result**

| **Dimension of Each Study:** | | | | | | | |
| --- | --- | --- | --- | --- | --- | --- | --- |
|  | GSE7621 | GSE8397A | GSE8397B | GSE20141 | GSE20186 | GSE20295 | GSE20333 |
| Common Genes | 1204 | 1204 | 1204 | 1204 | 1204 | 1204 | 1204 |
| Samples | 25 | 39 | 39 | 18 | 28 | 29 | 12 |
| **Quality Control Result:** | | | | | | | |
| Study | IQC^a^ | EQC^b^ | CQCg^c^ | CQCp^d^ | AQCg^e^ | AQCp^f^ | Rank |
| GSE8397-A | 6.45 | 0.64* | 307.65 | 55.12 | 1.48* | 3.26 | 2.08 |
| GSE8397B | 2.69 | 0.46* | 307.65 | 50.88 | 1.75* | 5.7 | 3.25 |
| GSE20333 | 4.66 | 1.68* | 11.96 | 25.03 | 0.49* | 2.36 | 3.5 |
| GSE20186 | 3.36 | 0.59* | 17.87 | 15.35 | 0.46* | 7.27 | 4 |
| GSE20141 | 2.2 | 0.63* | 7 | 32.29 | 0.34* | 6.54 | 4.67 |
| GSE7621 | 2.29 | 0.62* | 15.32 | 17.74 | 1.05* | 2.22 | 4.83 |
| GSE20295 | 2.91 | 1.16* | 2.7 | 1.18* | 0.16* | 0.53* | 5.67 |

^a^IQC: internal quality control; ^b^EQC: external quality control; ^c^CQCg: consistency quality control in genes; ^d^CQCp: consistency quality control in pathways; ^e^AQCg: accuracy quality control of differentially expressed gene detection; ^f^AQCp: accuracy quality control of pathway identification.
